# Special Issue: NextGen Materials for 3D Printing

**DOI:** 10.3390/ma11040555

**Published:** 2018-04-04

**Authors:** Chee Kai Chua, Wai Yee Yeong, Jia An

**Affiliations:** Singapore Centre for 3D Printing, School of Mechanical and Aerospace Engineering, Nanyang Technological University, Singapore 639798, Singapore; wyyeong@ntu.edu.sg (W.Y.Y.); anjia@ntu.edu.sg (J.A.)

**Keywords:** 3D printing, additive manufacturing, 4D printing, composite, smart materials, biomaterials, concrete, ceramic, metal

## Abstract

Only a handful of materials are well-established in three-dimensional (3D) printing and well-accepted in industrial manufacturing applications. However, recent advances in 3D printable materials have shown potential for enabling numerous novel applications in the future. This special issue, consisting of 2 reviews and 10 research articles, intends to explore the possible materials that could define next-generation 3D printing.

Three-dimensional (3D) printing or additive manufacturing (AM, also known as rapid prototyping) was invented three decades ago and has been evolving over the years in four fundamental respects: design, material, process, and application [1]. Among the four, material has long been a hurdle for AM to have a wider application in the manufacturing industry. Until today, only a handful of materials are well-established in 3D printing and well-accepted in industrial manufacturing applications. However, in recent years, many materials, most deemed as not printable before, have surfaced in the field as printable materials, for example, concrete [2,3], composite [4,5], shape-memory materials [6,7], and biological cells [8,9]. Though not many have reached the stage of commercialization, the endless possibilities of 3D printing have been undoubtedly manifested by these new materials. This has motivated us to think about what 3D printing materials could be tomorrow and hence we proposed a Special Issue on Next Generation Materials for 3D Printing.

This special issue covers 2 reviews and 10 research articles. First of all, Chang et al. present an overview of material strategies for developing next-generation 3D printing [10]. Yan et al. present a review on grain structure control of 3D-printed metallic materials [11]. In research articles, Bos et al. report the reinforcement effect of metal cable in 3D-printed concrete [12]. Chang et al. present the work of selective laser sintering of porous silica with a carbon additive [13]. It is found that the carbon additive improves the absorptivity of silica powder to the fiber laser. Scheithauer et al. report the successful fabrication of zirconia components with varying microstructures using thermoplastic suspensions with different contents of pore-forming agents [14]. Steyrer et al. present the influence of photoinitiator selection on the thermomechanical properties of various tough photopolymers [15]. Habib et al. show that an alginate-carboxymethyl cellulose hydrogel has good printability and improved mechanical integrity [16]. Huang et al. report the effect of hydroxyapatite and β-tri-calcium phosphate in polycaprolactone-ceramic composite scaffolds [17]. Khatri et al. present the development of a new functional composite with a soft magnetic property [18]. Teoh et al. show that the design and four-dimensional (4D) printing of a cross-folded origami structure is feasible and may have the potential for developing smart paper [19]. Finally, Wang et al. present the effect of T2 heat treatment on the microstructure and properties of selective laser-melted aluminum alloy samples [20].
